# Clericuzio-type poikiloderma with neutropenia and leg ulceration

**DOI:** 10.1016/j.jdcr.2023.08.045

**Published:** 2023-09-27

**Authors:** Atheer Al Haddabi, Ghulam Mufti, Anthony du Vivier, Tanya Nandini Basu

**Affiliations:** a Department of Dermatology, King’s College Hospital, London, United Kingdom; b Department of Haematology, King’s College Hospital, London, United Kingdom

**Keywords:** Clericuzio-type poikiloderma with neutropenia, pyoderma gangrenosum

## Introduction

We report a case of leg ulceration secondary to pyoderma gangrenosum (PG) in a patient with Clericuzio-type poikiloderma with neutropenia (PN). This is an autosomal recessive genodermatosis caused by loss of function mutations in the gene USB1 (on chromosome 16q21), which was first described by Clericuzio et al.^1^ The gene encodes a conserved phosphodiesterase enzyme that regulates the stability of spliceosomal U6-RNA and plays a key role in neutrophil maturation, and is expressed in skin keratinocytes.

## Case report

An 18 year old man presented during the COVID-19 pandemic with a 4 month history of a painful, enlarging ulcer on the right lower portion of the leg following impact from a shopping trolley. Our patient works at a supermarket. He had recently been diagnosed with PN. He took monthly testosterone subcutaneously for hypogonadism and underwent laser therapy, aged 9 years, for facial telangiectasia.

**Fig 1:**
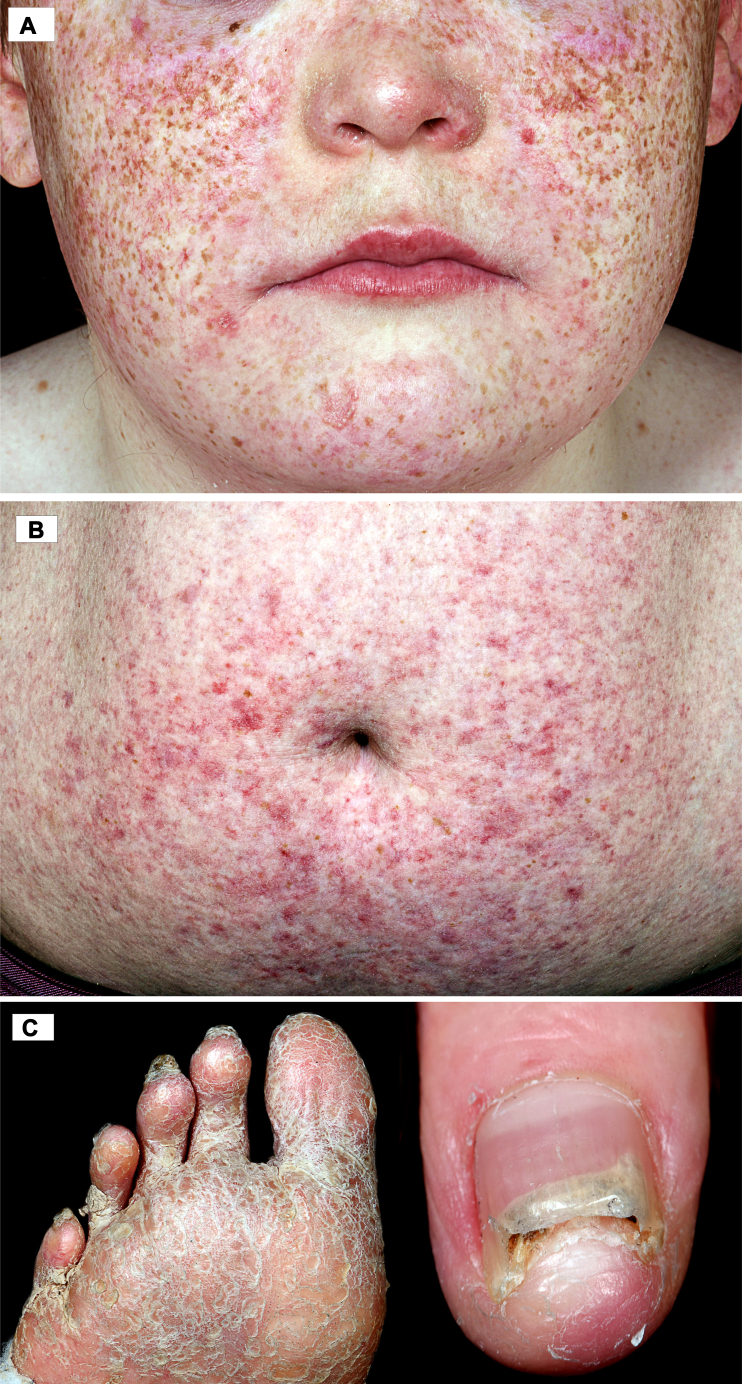
Highlights the classical cutaneous features of Clericuzio-type poikiloderma with neutropenia. **A,** Distinctive facies with a depressed nasal bridge, midfacial hypoplasia and poikiloderma (telangiectasia, atrophy, and hypo/hyperpigmentation). Our patient also had striking freckling. **B,** The periumbilical skin illustrates further widespread telangiectasia and hypopigmentation. **C,** The soles of the feet show hyperkeratosis and desquamation. Nail dystrophy and onycholysis affecting the hands.

**Fig 2:**
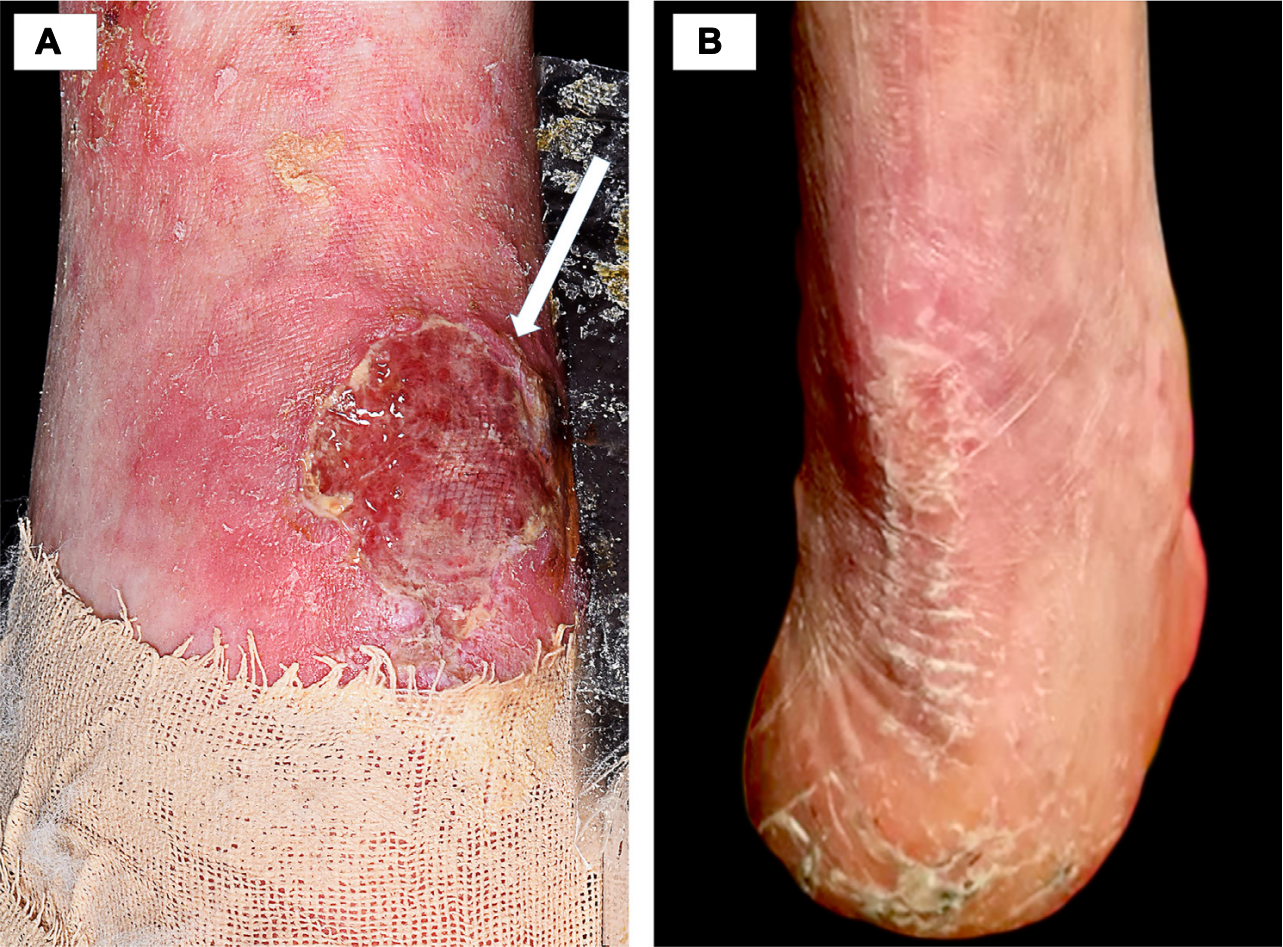
Pyoderma gangrenosum. **A,** A tender superficial 3 × 3 cm ulcer overlying the Achilles tendon on the right side of the heel with an undermined edge at the superior margin (shown by the *arrow*) consistent with pyoderma gangrenosum. **B,** Resolution of the ulcer after treatment with a tapering course of oral corticosteroids.

On examination, he had a short stature, a depressed nasal bridge and widespread poikiloderma with reticulated hypo/hyperpigmentation, atrophy, and telangiectasia, involving the face, trunk, and limbs (Fig 1, *A* and *B*). Striking palmoplantar hyperkeratosis and nail dystrophy were apparent (Fig 1, *C*). A 3 × 3 cm well-circumscribed tender ulcer with an undermined, overhanging violaceous edge overlay the right Achilles tendon (Fig 2, *A*). A full blood count confirmed neutropenia, of 0.85 × 10^9^/L (normal range 2.2-6.3 × 10^9^/L). Bacterial swabs from the ulcer showed no growth.

Aged 2 years, our patient was diagnosed with Rothmund-Thomson syndrome. Aged 15 years, the diagnosis was revised to Clericuzio-type poikiloderma when neutropenia prompted genetic testing. This revealed a homozygous c.673C>T USB1 gene mutation. Both nonconsanguineous parents were carriers.

He remains under hematological follow-up for neutropenia.

The ulcer on the right lower portion of the leg was clinically consistent with PG. Lending further weight to this diagnosis, the ulcer continued to extend despite excellent supportive treatment with nonadhesive dressings and compression bandaging at his local hospital. The patient declined a skin biopsy, as his skin heals poorly after minor injury (pathergy). Hence, a trial of empirical treatment for PG was instigated, with topical clobetasol propionate ointment twice daily and oral doxycycline 100 mg daily; this led to partial improvement in the ulcer (Fig 2, *B*). Despite the neutropenia and risk of COVID-19, oral corticosteroid therapy was commenced (prednisolone 30 mg daily, weaned by 5 mg every 3 days); the ulcer responded dramatically and healed completely within 3 weeks and remains clear 2 years later.

## Discussion

PN, first becomes apparent when infants (aged 6-12 months) develop an acute inflammatory papular rash that settles leaving widespread poikiloderma (a triad of skin atrophy, dyspigmentation, and telangiectasia) that is not photodistributed, followed by palmar plantar hyperkeratosis, calcinosis cutis, and nail dystrophy. Patients have distinctive facies with midfacial hypoplasia; alopecia is not reported. Noncyclical neutropenia is a hallmark, leading to recurrent sinopulmonary infections (aged <24 months). Nonhealing ulcers occur in later life. Cancer susceptibility to myelodysplasia, acute myeloid leukemia and cutaneous squamous cell cancers, means that patients require careful monitoring. Further features include, hypogonadotropic hypogonadism with delayed puberty, osteopenia, and decreased bone density.^2^

Patients with PN are sometimes erroneously diagnosed with Rothmund-Thomson syndrome where poikiloderma develops more gradually within the first 2 years of life. It is caused by mutations in a different gene, the RECQL4 gene which encodes a DNA helicase. There are discriminating clinical signs: in Rothmund-Thomson syndrome, the poikiloderma is photodistributed, there is alopecia with juvenile cataracts and no neutropenia. Other clinical findings include skeletal abnormalities such as short stature and radial ray anomalies. Patients with Rothmund-Thomson are also susceptible to malignancy including cutaneous squamous cell cancers, but, unlike patients with PN, are predisposed to osteosarcomas. Other genetic conditions with poikiloderma include dyskeratosis congenita, and with telangiectasia include Bloom and Werner syndromes.^3^^,^^4^

Skin ulcers that are slow to heal are associated with PN. However, a link between PG and Clericuzio-type poikiloderma has not been documented before. Of note, Altunay et al^5^ did report a case of a treatment-refractory cutaneous ulcer associated with presumed Rothmund-Thomson syndrome. This patient’s ulcer occurred after trauma and did not heal despite surgical dressings, antibiotics, and hyperbaric oxygen. Strikingly, this patient’s diagnosis was also later revised to be Clericuzio-type PN following genetic testing,^5^ as in our case. Moreover, the reported clinical features of the ulcer were notably similar to our patient. We speculate this ulcer may also have been superficial ulcerative PG.

Although PG is an autoinflammatory neutrophilic dermatosis, counterintuitively it can be associated with neutropenia in peripheral blood, as well as other myeloid blood dyscrasias such as myelodysplastic syndrome, acute myeloid leukemia, severe congenital neutropenia, and cyclical neutropenia.^6^^,^^7^ The pathogenesis of PG, with or without the context of peripheral neutropenia, is not well elucidated, but aberrant neutrophil trafficking is thought to play a role, associated with oscillating levels of neutrophil integrin expression (the cell surface molecules that play a key role in neutrophil migration by binding to the extracellular matrix).^8^

The prognosis for our patient remains unclear. In PN, only a subset of the 17 causative mutations identified so far, seems to predispose to developing cancer. The following USB1 mutations have been associated with malignancy; c.[531delA], c.[243G>A], c.[541C>T], and c.[502A>G]. This subset does not include our patient’s mutation. Thus, the causative pathogenic variant should be considered when managing these patients. The long-term follow-up of patients with identical USB1 genotypes is vital to help direct future monitoring for potential dermatological and hematologic malignancies.^9^^,^^10^ In our patient, it remains to be seen whether or not myelodysplasia will emerge and he remains under close hematological follow-up.

Our case highlights the importance of considering Clericuzio-type poikiloderma in a child with neutropenia and nonphotodistributed poikiloderma. This rare diagnosis is often missed initially. In such patients, before assuming that a slow to heal ulcer is mechanical due to skin fragility, it is important to entertain a diagnosis of PG, as appropriate treatment may enable wound healing. To our knowledge, this is the first report of PG observed in the context of this congenital PN.

## Conflicts of interest

None disclosed.
